# Choosing an insulin injector by a structured, pharmaceutical-neutral curriculum via an informed shared decision-making process in 349 insulin-naive patients with diabetes mellitus

**DOI:** 10.1007/s00592-023-02069-0

**Published:** 2023-04-02

**Authors:** Bernardo Mertes, Sybille Gödde, Christof Kloos, Nadine Kuniß

**Affiliations:** 1 Department for Diabetology, Centre for Cardioangiology Bethanien, AGAPLESION Hospital Bethanien, Frankfurt, Main, Germany; 2 Department of Internal Medicine III, Endocrinology and Metabolic Disorders, Jena University Hospital, Am Klinikum 1, 07740 Jena, Germany; 3 Outpatient Healthcare Centre Dr. med. Kielstein, Erfurt, Germany

**Keywords:** Pharmaceutical-neutral curriculum, Informed shared decision making, Insulin injector

## Abstract

**Background:**

The national guideline for diabetes type 2 claims to involve patients in their decision-making on therapy. Unfortunately, no structured, pharmaceutical-neutral curriculum is available to guide patients in this shared decision-making (SDM) process regarding the insulin injector. The aim of the study was to evaluate which injector patients chose after SDM process and the reasons for their choice.

**Methods:**

We developed a curriculum for a SDM process to choose an insulin injector for insulin-naive patients with diabetes mellitus, which took place immediately before the start of the initial treatment with insulin. It was conducted by a physician or diabetes educator with no conflicts of interest. All available human short-acting disposable insulin injectors (A, B and C) were handed out for try-out accompanied by individual counselling. The patients selected their injector of choice and were asked immediately afterwards about the criteria for their selection.

**Results:**

349 consecutive patients (94% diabetes type 2; age 58.6 + 13.4y; HbA1c 10.4 + 2.1%) were included. Patients choose Injector A in 10.0%, B in 61.9% and C in 28.1%. Criteria for selection were: design (41.8%), general impression (23.5%), dose window (7.7%), dose selection dial (7.4%), most practical (6.6%) and other (13%). Selection of a specific injector was not associated with age, diabetes type, diabetes duration, BMI, HbA1c, presence of concomitant diseases, retinopathy, neuropathy, diabetic foot or physician/diabetes educator.

**Discussion:**

Insulin-naive patients with diabetes mellitus chose their own insulin injector within a newly developed structured SDM process to meet the national guideline. Main selection criteria were design and practicability.

## Introduction

In Germany, the national guideline for diabetes type 2 claims to involve patients in their decision-making on therapy [1]. This is important to ensure the acceptance of the therapy, which persons with diabetes often have to continue for their lifetime. Thus, informed and shared decision making, for example, which therapy strategy is to be applied, is pivotal in clinical care. To increase therapy adherence, the type of insulin therapy (e.g. therapy with mixed insulin or short-acting insulin) must be suited and fit in the patient's life.

But even details that are completely unimportant from a medical point of view, such as the colour, the shape or the handling of the insulin injector, could improve the acceptance of the therapy. On the other hand, this arises the question who, if not the patient, decides which insulin injector to take and why. There are no systematic studies on this issue. A study conducted by Eli Lilly® compared preferences between two reusable insulin pen devices in pen-naïve adults with diabetes mellitus type 1 (DM1) and type 2 (DM2) in the United States of America [2]. Handling of the pen was the most common reason for choosing.

In Germany, there are three human short-acting disposable insulin injectors from different manufacturers. The insulins have identical pharmacodynamics properties, but the pens look different in terms of colour and shape. Handiness and handling also differ. Unfortunately, no structured, pharmaceutical-neutral curriculum is available to guide patients in this shared decision-making (SDM) process regarding the injector.

The aim of the study was to evaluate which injector patients chose after SDM process and the reasons for their choice.

## Research design and methods

Between 03/2019 and 12/2020, 349 patients with DM from an outpatient center for diabetology at secondary care level were included in the study. All patients were off their individual HbA1c target and initiation of insulin therapy was indicated.

Inclusion criteria were diagnosis of DM1 or DM2 with necessity of insulin therapy, the patient's willingness to independently implement insulin therapy and outpatient treatment as part of regular care at our clinic. Exclusion criteria were refusal or inability to participate.

### Intervention

We developed a curriculum for the SDM process to choose an insulin injector for insulin-naive patients with DM, which took place immediately before the start of the initial treatment with insulin. The teaching lesson was conducted by a physician or diabetes educator with no conflicts of interest. All available human short-acting disposable insulin injectors (A, B and C) were handed out for try-out accompanied by individual counselling. First, differences of the injectors were explained in terms of colour, shape, insulin dosage scale (black numbers on a white background or vice versa), click sounds (by setting the dose) and origin of the manufacturer (NovoNordisk/Denmark, Lilly/USA, Sanofi Aventis/Germany). The three insulin injectors were placed on the table at the same time and were always presented in the same sequence. It was then emphasised that the three insulins are identical in their effect. The patients were able to hold the three insulin injectors in their hands and try them out (dose selection dial and plunger button). The patients could independently select their injector and were asked immediately afterwards about the criteria for their selection („Why did you choose this injector? “). The SDM process lasted 5 min approx. Afterwards, the correct insulin injection was explained to the patients based on their individual selected insulin injector.

### Statistical analyses

Statistical analyses were performed with SPSS 25 (IBM Corporation, Armonk, NY, USA). All continuous data are presented as mean ± standard deviation (SD). Categorical data are described by absolute and relative frequencies. A regression analysis was used to assess the influence of covariates (e.g. age, diabetes duration) on choice of insulin injector. Significance was defined at the 0.05 level.

## Results

349 consecutive patients were included. The characteristics of the participants are shown in Table 1.

**Table 1:** Characteristics of the study participants and differences between choices of insulin injector

	All participants n = 349	Insulin injector			p-value*
		A	B	C	
		n = 35 (10.0%)	n = 216 (61.9%)	n = 98 (28.1%)	
Female, n (%)	108 (30.9)	8 (22.9)	67 (31.0)	33 (33.7)	0.493
Age (years)	58.6 ± 13.5	55.4 ± 14.7	58.4 ± 12.9	60.0 ± 14.1	0.211
Diabetes type, n (%)					
type 1	10 (2.9)	3 (8.6)	3 (1.4)	4 (4.1)	0.079
type 2	327 (93.7)	32 (91.4)	203 (94.0)	92 (93.9)	
unclear	12 (3.4)	0 (0)	10 (4.6)	2 (2.0)	
Diabetes duration (years)	7.2 ± 8.2	7.5 ± 6.5	7.1 ± 8.1	7.3 ± 9.0	0.950
BMI (kg/m^2^)	31.1 ± 7.2	30.4 ± 5.2	31.4 ± 7.9	30.7 ± 6.1	0.573
HbA1c (%, mmol/mol)	10.4 ± 2.1	9.9 ± 1.8	10.5 ± 2.3	10.4 ± 1.9	0.391
	90.2 ± 23.2	85.2 ± 19.3	90.9 ± 24.7	90.5 ± 21.0	
eGFR (ml/min)	113.3 ± 47.4	115.6 ± 43.2	114.0 ± 48.1	112.9 ± 47.5	0.965
Complications, n (%)					
Hypertension	253 (72.5)	21 (60.0)	161 (74.5)	71 (72.4)	0.203
Coronary heart disease	55 (15.8)	2 (7.4)	33 (15.3)	20 (20.4)	0.117
Polyneuropathy	105 (30.1)	10 (28.6)	62 (28.7)	33 (33.7)	0.659
Diabetic foot syndrome	9 (2.6)	0 (0)	7 (3.2)	2 (2.0)	0.493

^*^ between Insulin injector A, B and C

Participants chose Injector A in 10.0%, B in 61.9% and C in 28.1%. Criteria for selection were (Table 2): design (41.8%), general impression (23.5%), dose window (7.7%), dose selection dial (7.4), practicability (6.6%) and other reasons (13%). Manufacturer and production site was not a frequently cited reason (3.7%).

**Table 2:** Reasons for choosing insulin injector

	Study participants (total n = 349)
Design (colour, shape, weight, thickness), n (%)	146 (41.8)
General impression, n (%)	82 (23.5)
Dose window (scale), n (%)	27 (7.7)
Dose selection dial (click sound, setting technique), n (%)	26 (7.4)
Most practical, n (%)	23 (6.6)
Design and Dose window, n (%)	16 (4.6)
Manufacturer/production site, n (%)	13 (3.7)
Design and Most practical, n (%)	10 (2.9)
Family members also have this pen, n (%)	4 (1.1)
Looks more solid, n (%)	2 (0.6)

Selection was not associated with age, diabetes type, diabetes duration, BMI, HbA1c, presence of concomitant diseases, retinopathy, neuropathy, diabetic foot or physician/diabetes educator. Table 1 shows differences between choices of insulin injectors.

## Discussion

In this study, insulin-naive patients with DM chose their own insulin injector within a newly developed structured SDM process to meet the national guideline. This was performed in a clinical setting under real-life conditions without industry involvement and by a team of therapists with no conflicts of interest.

From a clinical point of view design and colour of the insulin injection does not matter for the choice. However, this does not reflect the patient's perspective. In addition, the fact that the patient was involved in the process to choose the injector may improve the adherence.

Patients have their own preferences and express them when they have the opportunity. The study participants seem to care which injector they finally end up with. If this improves the overall adherence to therapy or influences a clinically relevant endpoint cannot be concluded on the basis of this study and can only be clarified by further investigations.

The curriculum for the selection of the injector by the patient has been easily feasible and applicable under everyday clinical conditions. The curriculum empowers the patient to choose his injector without being biased and without being patronized by medical staff in an objective and rapid manner.

Strengths of this study are the implementation under real-life clinical conditions with a high number of participants. Another strength is the restriction to insulin naïve patients who had not previously been influenced by the use of an injection. However, this study is limited by the fact that the criteria used by the patient to select the injection did not meet industry and market research standards. Furthermore, the sequence of the presentation of the insulin injectors was always the same. This process may have influenced the decision.

## Conclusions

Insulin-naive patients with DM chose their own insulin injector using a newly developed structured SDM process requested in the national guideline. Main selection criteria were design and practicability.

## Abbreviations

BMI: Body mass index
DM1: Diabetes mellitus type 1
DM2: Diabetes mellitus type 2
